# Measuring HIV-related mortality in the first decade of anti-retroviral therapy in sub-Saharan Africa

**DOI:** 10.3402/gha.v7.24787

**Published:** 2014-05-21

**Authors:** Jim Todd, Emma Slaymaker, Basia Zaba, Mary Mahy, Peter Byass

**Affiliations:** 1 London School of Hygiene and Tropical Medicine, Keppel Street, WC1E 7HT, London, UK; 2 Epidemiology Section, UNAIDS, Geneva, Umeå Centre for Global Health Research, Department of Public; 3 Health and Clinical Medicine, Umeå University, Umeå, Sweden

For the past 30 years, many communities and countries in sub-Saharan Africa have suffered from the ravages of the HIV epidemic. During this time the mortality rates in HIV-infected people have been 10–20 times higher than in HIV-uninfected people (1). Since the advent of anti-retroviral therapy (ART), mortality and morbidity have decreased considerably (2). However, it has been difficult to get reliable estimates of the impact of ART in population-based studies. The measurement of the impact of ART on mortality requires long-term follow-up in communities where regular HIV testing allows the estimation of mortality by HIV status, such as the population cohorts in the network for Analysing Longitudinal Population-based HIV data in Africa (ALPHA) (3). An alternative estimation from verbal autopsy (VA) requires reliable tools and consistent interpretation of causes of death, which have been taken forward by recent standards from the World Health Organization (WHO) and the development of the corresponding InterVA-4 model for cause of death assignment (4, 5).

This supplement puts together evidence from different sources in order to estimate the impact of ART on adult HIV mortality. The papers in this supplement use different measures to estimate the effect of ART, and the stories from the different papers provide a consistent picture of the reduction in HIV-related mortality with the advent of ART in sub-Saharan Africa. However, in developed countries the mortality in HIV-positive people on ART is similar to the mortality in the general population (6), but based on the evidence from the population studies reported in this supplement, it is not the case in sub-Saharan Africa, even if it may be achieved in the future.

The AIDS impact module within the Spectrum software is a modeling tool for estimating many facets of the HIV epidemic in the population, usually using HIV prevalence and program data available at the national level (7). We can evaluate the mortality estimate from the Spectrum model against the empirical data from longitudinal studies if we can accurately create a Spectrum model for the small area where the longitudinal study is located. This comparison allows us to validate the assumptions used in Spectrum that lead to the mortality estimates from the model.

The paper by Kanjala et al. documents the changes in age-specific mortality rates (ASMR) from 1994 to 2010 in a cohort in Tanzania (8). This study showed an overall reduction in mortality in those aged 15–59 from 12.3 per 1,000 person years (95% CI 11.5–13.1) in the 5 years before ART was introduced to 8.0 per 1,000 person years (95% CI 7.2–8.8) after ART was introduced. In that period, the greatest reduction was seen in HIV positives aged 30–45 years, with a 44% reduction in male mortality, and a 71% reduction in female mortality in this group. For both sexes, the HIV-attributable mortality among the population showed a reduction from more than 50% in 2000, to around 35% in 2010, which can be related to the availability of ART since around 2005. The pooled analysis of adults aged 15–54 in five sites in East and Southern Africa showed an overall halving of the excess mortality in HIV positives, with the decline evident across all sites and for both sexes (9).

Four papers in this supplement reported the use of InterVA-4 to interpret the cause of death from VA data. Byass et al. validated the InterVA-4 model against pre-mortem serological data from six ALPHA sites (5). This showed 90% specificity in identifying HIV-related deaths among those with confirmed HIV sero-status, which was consistent across the six sites, and across time, making InterVA-4 an effective tool in assessing HIV-related mortality. Glynn et al. compared the use of InterVA-4 with the interpretation of deaths by clinician review in Malawi from 2002 to 2012 (10). The results confirm the specificity of InterVA-4, as 88% of the deaths identified as unrelated to HIV by the physicians were correctly identified by InterVA-4. For HIV-related deaths defined by the physician review, InterVA-4 identified 59% due to HIV/AIDS, and a further 20% where TB was the cause of death. Byass et al. identified these two causes (HIV/AIDS and TB) plus acute respiratory infections as highly associated with HIV positivity, indicating that these are likely causes of death in people living with HIV (5). Both Glynn and Kanjala reported that InterVA-4 may underestimate the number of deaths due to HIV, perhaps through coding of such deaths under different causes (8, 10). This raises questions about the ICD-10 coding rules whereby almost all deaths among HIV positives are expected to be coded as HIV related (11). This is likely to become a bigger issue as more HIV-positive individuals experience ART for longer periods of time, before going on to die from a potentially wider range of causes.

Two papers used Spectrum model estimates and showed good agreement with empirical data in Kenya and Tanzania (12, 13). Oti et al. compared the Spectrum model outputs for the Nairobi area against the health and demographic surveillance site (HDSS) observed mortality using InterVA-4 to interpret cause of death. The Spectrum model estimated that in 2003, 63% of adult mortality was HIV-related, decreasing to 40% in 2010, while for InterVA-4, including deaths from both HIV/AIDS and TB showed that 59% were HIV-related in 2003, and 46% in 2010. In Tanzania, using adult mortality between the ages of 15 and 49 years, Michael et al. found the Spectrum model estimated that HIV-related mortality had fallen from 43% in 1994 to 37% in 2009, compared to the results from the demographic and serological data which showed a reduction from 39 to 22% over the same period. They concluded that Spectrum predicts a greater proportion of adult deaths being due to HIV than observed in the cohort, and speculate that this may have been influenced by the low reported uptake of ART services in the cohort. Further work is needed to refine the Spectrum models created using small area data (as opposed to national data which is usually used for Spectrum estimates), but this seems a useful challenge to bring together the Spectrum model with existing HDSS data, and to identify the limitations of such comparisons.

Most countries have now adopted the 2010 WHO guidelines to initiate ART for those with HIV infection and CD4 counts under 350 cells per mm^3^, but new guidelines in 2013 recommend initiation of ART in all those with CD4 counts less than 500 cells per mm^3^ (14). Masiira et al. used standardized mortality rates (SMR) for HIV-positive, ART-naive Ugandan adults, and compared the mortality of those with CD4 counts between 350 and 499 cells per mm^3^, to those with CD4 counts greater than 500 cells per mm^3^, and with the Ugandan general population (15). Mortality rates were 1.6 times higher in those with lower CD4 counts (between 350 and 499 cells per mm^3^), and 2.5 times higher than the general population. The excess HIV mortality in those with CD4 counts between 350 and 499 cells per mm^3^ would be prevented with the implementation of the WHO guidelines for people living with HIV in developing countries.

The final paper in the supplement, by Levira et al., looks at a different impact that ART may have on mortality (16). Many people migrate back from the cities to their rural home villages when severely sick and expecting to die (17). With the advent of ART, the mortality among urban–rural migrants in Tanzania has reduced by 39% compared to a reduction of 27% among non-migrants.

The message from the papers in this supplement across the first decade of ART roll-out in sub-Saharan Africa shows a consistent reduction of overall mortality rates in the population of around 30%, with the proportion of deaths attributable to HIV falling by 30–50%. In this period, excess mortality among HIV-positive adults has halved, but the mortality rates in HIV positives are still up to 10 times higher than among HIV negatives (9). In the coming decade, there is a lot more to be done in terms of increasing access to, and availability of, ART. This should lead to further reductions in mortality rates, but will also bring new challenges to measuring HIV-related mortality, particularly among those who have received long-term treatment.

*Jim Todd* *Emma Slaymaker* *Basia Zaba* London School of Hygiene and Tropical Medicine, Keppel Street WC1E 7HT, London, UK *Mary Mahy* Epidemiology Section, UNAIDS, Geneva Umeå Centre for Global Health Research Department of Public*Peter Byass* Health and Clinical Medicine, Umeå University Umeå, Sweden
